# Abdomen Depth and Rectus Abdominis Thickness Predict Surgical Site Infection in Patients Receiving Elective Radical Resections of Colon Cancer

**DOI:** 10.3389/fonc.2019.00637

**Published:** 2019-07-15

**Authors:** Song Liu, Meng Wang, Xiaofeng Lu, Min Feng, Feng Wang, Liming Zheng, Wenxian Guan

**Affiliations:** ^1^Department of Gastrointestinal Surgery, Nanjing Drum Tower Hospital, The Affiliated Hospital of Nanjing University Medical School, Nanjing, China; ^2^Department of General Surgery, Drum Tower Clinical Medical College, Nanjing Medical University, Nanjing, China

**Keywords:** surgical site infection (SSI) risk, colon cancer, rectus abdominis, subcutaneous fat thickness, abdomen depth

## Abstract

**Background:** Surgical site infection (SSI) hampers the advantages of surgical management, which requires early forecast particularly in patients receiving colorectal surgery. This study is to explore potential relationship between individual abdominal anatomic characteristics including subcutaneous fat thickness (SFT), rectus abdominis thickness (RAT), and abdomen depth (AD), with the incidence of SSI in elective radical resection of colon malignancy.

**Materials and Methods:** This retrospective case-control study has recruited 55 patients in each SSI and non-SSI group with propensity score match method. Demographics, clinical attributes, and pre- and intra-operative information were compared between groups with univariate analysis to elicit significant parameters, which were subsequently brought into logistic regression and receiver-operating characteristic (ROC) analysis.

**Results:** Patients with SSI showed lower preoperative albumin (*p* = 0.0022), higher RAT (*p* = 0.014), AD (*p* = 0.029), and the multiplied value (RAT × AD) (*p* = 0.0026) contrasted with patients without SSI. Logistic regression demonstrated RAT × AD as an independent risk factor for SSI (OR = 1.007, *p* < 0.001) and a biomarker for SSI prediction (AUC = 0.83, 95% CI: 0.74 ~ 0.91).

**Conclusions:** Preoperative RAT and abdomen depth are associated with the risk of postoperative SSI in patients receiving elective radical resection of colon cancer.

**Trial Registration:** www.researchregistry.com, identifier researchregistry3669

## Introduction

Surgical site infection (SSI) is one of the most frequent healthcare-associated infections (1). Previous studies have shown that SSI results in delayed rehabilitation, prolonged hospital stay and long-term disability (2). It significantly hampers the benefits of surgical management in addition to reducing satisfaction from patients (3). Therefore, identification of risk factors of SSI for early intervention becomes valuable in clinical practice.

Colon cancer has become one of the most common worldwide malignancies (4). Elective radical resection is the most effective and essential method for colon cancer. However, SSI occurs frequently especially in colorectal laparotomy, of which the incidence is approaching 20% (5). Moreover, rectus abdominis incision is able to expose to elevated risk of SSI due to assumed more abundant blood supply in muscles for easier bacteria colonization.

Individual abdominal anatomic characteristics, including subcutaneous fat thickness (SFT), rectus abdominis thickness (RAT), and abdomen depth (AD) vary dramatically. Very few studies have investigated the effect of abdominal anatomic features on SSI development. Herein, we will conduct a retrospective case-control study with propensity score match to identify risk factors for SSI and evaluate their abilities in SSI prediction in patients receiving elective radical resection of colon cancer.

## Materials and Methods

### Patient Selection

From Jan 2015 to Dec 2017, Patients with colon cancer that registered in Gastrointestinal Surgery of our hospital were screened for qualification. The inclusion criteria included: (1) definitive pathological diagnosis of colon cancer (including ascending, transverse, descending and sigmoid colon cancer); (2) elective instead of emergent radical resection of colon cancer; (3) initial laparotomy instead of laparoscopy or reoperation; (4) rectus abdominis incision instead of midline (linea alba) incision; (5) the availability of preoperative CT scan; (6) definitive SSI with positive incisional secretion culture result in SSI group; (7) completeness of demographic, pre- and intra-operative, and pathologic data.

Demographics, clinical information, preoperative lab result, surgical information and pathology were collected from the Electronic Medical Record System. SFT, RAT, and AD are all measured at the umbilicus level of supine CT images (Figure S1). SFT is defined as the largest sagittal distance between the parietal and visceral sides of subcutaneous fat. RAT is defined as the largest sagittal distance between the parietal and visceral sides of rectus abdominis. AD is defined as the sagittal distance between the bottom of umbilicus and top of vertebra. CT measurements of the three parameters are made in triplicate by three independent operators, and the mean value is accepted for further analysis. SSI in the current study was defined according to WHO criteria (6). An infection that appeared at the surgical site within 30 postoperative days and that was characterized by any of the following circumstance: purulent drainage from surgical site, organism cultured from the fluid of surgical site, and/or incisional inflammation (pain, tenderness, localized swelling, and redness). All three subtypes of SSI including superficial, deep and organ/space SSI were taken into account for subsequent analysis.

All qualified patients were then divided into SSI or non-SSI group according to the incisional outcome. The baseline between two groups in the entire cohort were initially compared, and propensity score matching was subsequently performed using the corresponding module in SPSS software (version 23.0; SPSS Inc., Chicago, IL) to yield two comparable groups for further analysis.

### Perioperative Management

All participants received preoperative management including preoperative nutritional support by enteral (if bowel obstruction was absent) and/or parenteral nutrition (if bowel obstruction existed), as well as reinforcement of physical rehabilitation. Prophylactic antibiotics (single dose of cefuroxime) was intravenously used within 30 min before surgery (7). Intraoperative re-administration of antibiotics was administrated in prolonged surgery or significant intra-operative loss of blood. All participants received elective radical laparotomy of colon cancer, i.e., for ascending/descending colon cancer, standard radical right/left hemicolectomy was performed; for transverse/sigmoid colon cancer, tumor removal with sufficient surgical margin, associated mesentery and draining lymph nodes removal together with subsequent one-stage anastomosis was performed. Incisional secretion culture was immediately performed in all suspicious SSI cases. Broad-spectrum antibiotics was prescribed when SSI occurred, and adjusted to sensitive anti-infective agents according to the specific culture results. Topical dressing, suture removal, pus drainage, debridement and even open-wound care would be carried out depending on the severity of infection. All surgical procedures were performed by the same team including two gastrointestinal consultants, four full-time attendings and four residents.

### Statistics

All analysis was 2 tailed. Statistical difference was considered when *p* < 0.05. Continuous variables were presented as mean ± SD (standard deviation) and analyzed using unpaired *t*-test with Welch's correction. Categorical variables were presented as frequency (percentage) and analyzed using chi-square with Fisher's exact test. All variables that have been found statistically different between groups would be entered into the logistic regression and ROC analysis. Binary logistic regression analysis with forward (conditional) stepwise selection was performed to identify significant risk factors for postoperative SSI. Receiver-operating characteristic (ROC) analysis was performed to evaluate prediction ability and optimal cut-off value of all biomarkers. All above analysis was performed within GraphPad Prism Software (version 7.0; GraphPad, San Diego, CA, USA) and SPSS software (version 23.0; SPSS Inc., Chicago, IL).

### Ethics

This study has been approved by the Ethics Committees of Nanjing Drum Tower Hospital (DTH-IRB-2014-032). As a retrospective study, informed content is not required from participants.

## Results

From Jan 2015 to Dec 2017, a total of 374 patients with colon cancer that received elective radical resection were enrolled into this study. According to the occurrence of SSI, 73 patients were distributed into SSI group and the other 301 patients were distributed into non-SSI group. The overall incidence of SSI was 19.5%. All infections improved significantly, and no patient deceased from SSI. Male predominance and middle-age pattern were observed in both groups, although patients in SSI group were significantly older than those in non-SSI group (*p* = 0.023). Patients in SSI group exhibited higher BMI (*p* = 0.027), more past abdominal surgical histories (*p* = 0.0091), more tobacco usage (*p* = 0.0001), and more comorbidities (*p* = 0.011) compared to non-SSI group. The most frequent location of tumor was ascending colon in SSI group and sigmoid colon in non-SSI group, respectively (*p* = 0.0024). Patients in SSI group demonstrated advanced tumor stage compared to those in non-SSI group (*p* = 0.0001) (Table 1).

**Table 1:** Clinical characteristics of patients between SSI and non-SSI group.

	**Entire cohort (*****n*** **=** **374)**			**Propensity-matched cohort (*****n*** **=** **110)**		
	**SSI (*n* = 73)**	**Non-SSI (*n* = 301)**	***p***	**SSI (*n* = 55)**	**Non-SSI (*n* = 55)**	***p***
Male (*n*, %)	49 (67.1%)	191 (63.4%)	0.79	36 (65.4%)	34 (61.8%)	0.84
Age (years)	68.3 ± 7.8	60.4 ± 8.3	0.023^*^	66.9 ± 11.6	64.3 ± 11.1	0.24
BMI (kg/m^2^)	25.6 ± 3.9	22.3 ± 2.9	0.027^*^	24.2 ± 3.1	23.1 ± 3.8	0.53
Past abdominal surgical history (*n*, %)	30 (41.1%)	76 (25.2%)	0.0091^*^	15 (27.2%)	15 (27.2%)	1.00
Tobacco usage (*n*, %)	22 (30.1%)	32 (10.6%)	0.0001^*^	8 (14.5%)	6 (10.9%)	0.78
Comorbidity (*n*, %)			0.011^*^			0.78
Diabetes mellitus	15 (20.5%)	58 (19.2%)	–	11 (20.0%)	12 (21.8%)	–
Hypertension	30 (41.1%)	101 (33.5%)	–	22 (40.0%)	18 (32.7%)	–
Others	21 (28.8%)	28 (9.3%)	–	4 (7.3%)	5 (9.1%)	–
Tumor location (*n*, %)			0.0024^*^			0.16
Ascending	30 (41.1%)	73 (24.3%)	–	21 (38.2%)	15 (27.2%)	–
Transverse	6 (8.2%)	13 (4.3%)	–	4 (7.3%)	5 (9.1%)	–
Descending	9 (12.3%)	27 (9.0%)	–	5 (9.1%)	1 (1.8%)	–
Sigmoid	28 (38.4%)	188 (62.4%)	–	25 (45.5%)	34 (61.8%)	–
Tumor stage (*n*, %)			0.0001^*^			0.21
Stage I	8 (10.9%)	28 (9.3%)	–	6 (10.9%)	5 (9.1%)	–
Stage II	24 (32.9%)	179 (59.5%)	–	17 (30.9%)	26 (47.3%)	–
Stage III	41 (56.2%)	94 (31.2%)	–	32 (58.2%)	24 (43.6%)	–

* *Significant statistical difference*.

Subsequent propensity score matching was performed to establish two comparable groups due to the heterogenetic clinical characteristics in the entire cohort. Each group contained 55 qualified patients with statistically similar baseline (Table 1). Superficial (41.8%) and deep (47.3%) infections occupied the majority of SSI events. More than half of SSI was caused by Gram-negative bacteria, especially *Escherichia coli* (54.5%). The most common Gram-positive pathogens of SSI were *Enterococcus faecalis* (16.4%) and *Enterococcus faecium* (7.3%). The other pathogens included *Staphylococcus epidermidis, Streptococcus pneumonia, Citrobacter freundii, Acinetobacter baumannii*, etc. (Table 2).

**Table 2:** Characteristics of surgical site infections.

**Pathogen of infection**	**Type of infection**			**Total**
	**Superficial**	**Deep**	**Organ & Space**	
*Escherichia coli*	13	14	3	30 (54.5%)
*Enterococcus faecalis*	6	2	1	9 (16.4%)
*Enterococcus faecium*	2	2	0	4 (7.3%)
Others^*^	2	8	2	12 (21.8%)
Total	23 (41.8%)	26 (47.3%)	6 (10.9%)	55

* *Other pathogens include Staphylococcus epidermidis, Streptococcus pneumonia, Citrobacter freundii, Acinetobacter baumannii, etc*.

Preoperative lab results showed higher WBC, lower hemoglobin and higher C-reactive protein levels in propensity-matched SSI group. Nevertheless, a statistical difference of above parameters was not detected except serum albumin that was significantly lower in SSI compared to non-SSI group (*p* = 0.0022). Duration of operation and type of anastomosis were similar between two groups as well (Table 3).

**Table 3:** Pre- and intra-operative index of patients between SSI and non-SSI groups.

	**SSI (*n* = 55)**	**Non-SSI (*n* = 55)**	***p***
White blood cell (×10^9^/L)	6.6 ± 2.4	5.9 ± 1.7	0.14
Hemoglobin (g/L)	115.1 ± 20.6	121.4 ± 17.7	0.13
Albumin (g/L)	36.6 ± 3.9	39.2 ± 3.6	0.0022^*^
C-reactive protein (mg/L)	17.2 ± 21.2	11.0 ± 23.0	0.18
Duration of operation (min.)	187.2 ± 55.4	205.0 ± 60.0	0.11
Type of anastomosis (*n*, %)			0.35
End-to-end	16 (29.1%)	19 (34.5%)	–
End-to-side	33 (60.0%)	26 (47.3%)	–
Side-to-side	6 (10.9%)	10 (18.2%)	–

* *Significant statistical difference*.

The following three parameters were calculated to describe the abdominal anatomic features: the SFT, the RAT and the abdomen depth. Table 4 demonstrated that patients developed SSI with slightly higher SFT value, significantly higher RAT (*p* = 0.014) and AD (*p* = 0.029) values compared to controls. Moreover, the multiplied value (RAT × AD) was even more significantly different between SSI and non-SSI groups (*p* = 0.0026).

**Table 4:** Abdominal anatomic features of patients between SSI and non-SSI groups.

	**SSI (*n* = 55)**	**Non-SSI (*n* = 55)**	***p***
Subcutaneous fat thickness (mm)	19.2 ± 7.3	17.7 ± 7.5	0.31
Rectus abdominis thickness (RAT) (mm)	9.4 ± 2.7	8.1 ± 2.1	0.014^*^
Abdomen depth (AD) (mm)	82.3 ± 23.6	71.7 ± 23.8	0.029^*^
RAT × AD (mm^2^)	766.0 ± 304.4	589.1 ± 256.0	0.0026^*^

* *Significant statistical difference. All values are measured at umbilicus layer of CT images*.

**Figure 1:**
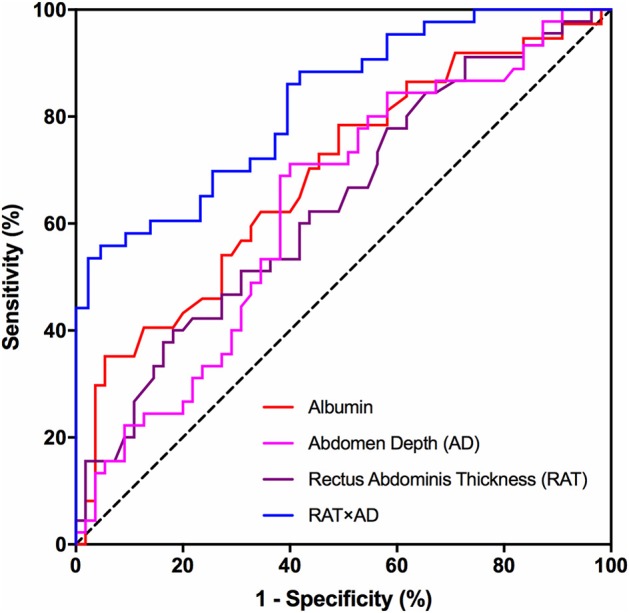
ROC analysis of risk factors for SSI prediction. Receiver-operating characteristic (ROC) curves for preoperative albumin, AD, RAT and the multiplied value of AD and RAT (RAT × AD) in predicting postoperative surgical site infections in patients receiving elective radical resection of colon cancer. RAT, rectus abdominis thickness; AD, abdomen depth.

Binary logistic regression analysis was subsequently performed to identify risk factors for SSI development. Significant parameters detected by previous univariate analysis, including albumin, RAT, AD, and RAT × AD, were brought into logistic regression model. Table 5 demonstrated that only RAT × AD was the significant risk factor for postoperative SSI in patients receiving elective radical resection of colon cancer (*p* < 0.001, OR = 1.007, 95% CI: 1.003 ~ 1.010). To investigate the predictive ability for SSI, the above four parameters were introduced into ROC analysis. Table 6 illustrated that RAT × AD exhibited the best performance in SSI prediction (AUC = 0.83, 95% CI: 0.74 ~ 0.91, cut-off = 716.1 mm^2^, sensitivity = 55.8%, specificity = 95.3%), compared to moderate abilities of the others with AUC ranging from 0.63 to 0.69 (Table 6; Figure 1).

**Table 5:** Risk factors for surgical site infection using logistic regression analysis.

	**95% CI**	**OR**	***P***
Albumin	–	–	0.055
Rectus abdominis thickness (RAT)	–	–	0.065
Abdomen depth (AD)	–	–	0.192
RAT × AD	1.003 ~ 1.010	1.007	<0.001

**Table 6:** Prediction of surgical site infection.

	**AUC (95% CI)**	Cut-off value^*^	**Sensitivity**	**Specificity**
Albumin	0.69 (0.58 ~ 0.80)	34.7 g/L	35.1%	94.6%
Rectus abdominis thickness (RAT)	0.64 (0.53 ~ 0.75)	9.91 mm	40.0%	81.8%
Abdomen depth (AD)	0.63 (0.53 ~ 0.74)	74.2 mm	71.1%	60.0%
RAT × AD	0.83 (0.74 ~ 0.91)	716.1 mm^2^	55.8%	95.3%

* *Cut-off value is yielded when the sum of sensitivity and specificity reaches maximum*.

## Discussion

Herein, we summarize our main findings of this study. By comparing 55 SSI and propensity score matched- 55 non-SSI patients receiving elective radical resection of colon cancer, we discovered that patients with preoperative lower albumin level, higher RAT, AD, and RAT × AD values augments the risk of SSI. Logistic regression identified that the multiplied value of RAT and AD (RAT × AD) is an independent risk factor for SSI. Further diagnostic power analysis confirmed that RAT × AD could serve as a biomarker for SSI prediction in these patients. These findings bring attentions to the effect of individual abdominal anatomic features on SSI development, and remind us to evaluate risk of SSI in patients with higher value of RAT and AD.

Surgical site infection is one of most common complications in general surgery. SSI leads to delayed rehabilitation and risk of incisional hernia. It is crucial to prevent SSI in patients with malignancies to accelerate postoperative recovery for subsequent potential conversion therapy. A bundle of measures has been established against postoperative SSI, including prophylactic antibiotics, skin preparation, aseptic operative instrument, and technique. Nevertheless, SSI is still occurring especially in major gastrointestinal surgery. Personal factors such as age, BMI, underlying disease, and nutritional status, as well as tumor stage, are all associated with the risk of SSI. We assume local factors such as thickness of subcutaneous fat and rectus abdominis, as well as depth of abdomen, can affect the rate of SSI in abdominal surgery. To exclude the potential cofounding effect of personal factors on incidence of SSI, we adopted propensity match to create homogeneity of baseline characteristics between two groups.

Albumin serves as a nutritional marker that has been suggested as a protective factor of postoperative complications in multiple diseases (3, 8, 9). Our study reveals difference of preoperative albumin between SSI and non-SSI groups in univariate model. However, albumin has lost the significance on multivariate model, implying underlying interactions between albumin and other parameters.

Thicker subcutaneous fat can lead to increased tension at suture line that is associated with less blood supply, and results in higher risk of incisional liquefaction and delayed wound healing. Previous studies have suggested a role of subcutaneous fat in the occurrence of SSI. Nakagawa et al. revealed that SFT is an independent risk factor for SSI in patients undergoing colorectal surgery (10), which is consistent with findings from Lee et al. (11) and Fujii et al. (12). Tongyoo et al. discovered that the subcutaneous thickness of abdominal wall is associated with SSI rate, especially in contaminated incisions (13). However, Kwaan et al. found that abdominal wall thickness is positively associated with incidence of SSI after colorectal surgery, but loses its significance on multivariate analysis (14). Furthermore, Osterhoff et al. concluded that SFT has no influence on the incidence of SSI (15). Our data demonstrated that patients in SSI group exhibited higher SFT, which is however not an independent risk factor for SSI and fails to serve as a biomarker for SSI prediction.

Thicker rectus abdominis can theoretically provide more abundant blood supply and thereby increases the possibility of bacteria colonization. All participants in this study received rectus abdominis incision, which highlights the importance of rectus abdominis in incisional healing. However, exclusion of midline (linea alba) incision, that is preferred in some areas during colon surgery, brings potential selection bias into this study.

Deeper abdomen leads to challenging surgical exposure and prolonged duration of surgery. It could also correlate with visceral adiposity since larger abdominal cavity could accommodate more visceral fat, which has been associated with multiple diseases (16). Indeed, a series of studies have clearly shown the statistical association between abdomen depth and visceral fat (17–20). Unfortunately, visceral adiposity was not measured by CT, waist/hip circumference ratio or bioelectrical impedance in this study. We failed to evaluate the correlation between abdomen depth, visceral adiposity and development of SSI. Meanwhile, few literature provides (patho-)/physiological correlation between abdominal depth and visceral adiposity.

To our knowledge, our study is the first investigation toward the role of RAT and abdomen depth in the occurrence of SSI. Our findings have highlighted the association between these two abdominal anatomic index and SSI development in a specific cohort of patients who undergo elective abdominal surgery via rectus abdominis incision.

We are fully aware of possible limitations. First of all, this is a single-center retrospective case-control study. Second, several potential risk factors (including intraoperative hypothermia, inadequate oxygenation and hyperglycemia) were not assessed in this study. Third, our study cannot provide mechanism by which abdominal anatomy affects the risk of infections. Nevertheless, our study has provided a comprehensive evaluation in the risk factors of SSI in elective radical resection of colon cancer. Future large prospective studies are awaited to provide strategies of SSI prevention in these patients.

## Conclusions

Preoperative RAT and abdomen depth correlate with the risk of postoperative SSI in patients receiving elective radical resection of colon cancer.

## Data Availability

The datasets generated for this study are available on request to the corresponding author.

## Ethics Statement

This study has been approved by the Ethics Committees of Nanjing Drum Tower Hospital (DTH-IRB-2014-032). As a retrospective study, informed content is not required from participants. The proof from the Ethics Review Board has been attached as a Supplementary File.

## Author Contributions

SL, LZ, and WG contributed conception and design of the study. SL organized the database. SL, MW, MF, FW, and XL performed the statistical analysis. SL and MW wrote the draft of the manuscript. All authors contributed to manuscript revision, read and approved the submitted version.

### Conflict of Interest Statement

The authors declare that the research was conducted in the absence of any commercial or financial relationships that could be construed as a potential conflict of interest.

## Supplementary Material

Figure S1 Illustration of measurement of abdominal anatomic characteristics. All parameters are measured at the umbilicus level of supine CT images. SFT is defined as the largest sagittal distance between the parietal and visceral sides of subcutaneous fat. RAT is defined as the largest sagittal distance between the parietal and visceral sides of rectus abdominis. AD is defined as the sagittal distance between the bottom of umbilicus and top of vertebra. CT measurements of the three parameters are made in triplicate by three independent operators, and the mean value is accepted for further analysis. SFT, subcutaneous fat thickness; RAT, rectus abdominis thickness; AD, abdomen depth.
