# Supplementary figures and images for: Abdomen Depth and Rectus Abdominis Thickness Predict Surgical Site Infection in Patients Receiving Elective Radical Resections of Colon Cancer
Source: Front Oncol. 2019 Jul 15;9:637. doi: 10.3389/fonc.2019.00637 (PMC6644599; doi:10.3389/fonc.2019.00637)

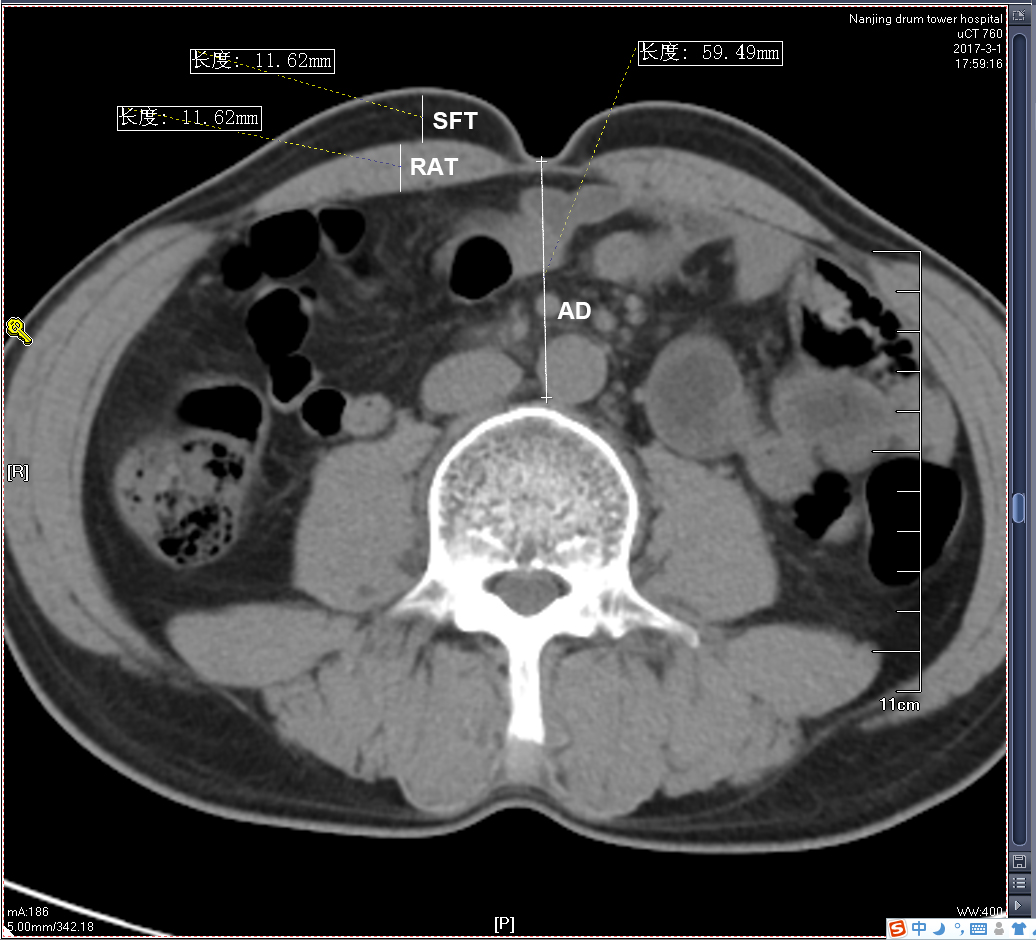

Supplement: Figure S1 — Illustration of measurement of abdominal anatomic characteristics. All parameters are measured at the umbilicus level of supine CT images. SFT is defined as the largest sagittal distance between the parietal and visceral sides of subcutaneous fat. RAT is defined as the largest sagittal distance between the parietal and visceral sides of rectus abdominis. AD is defined as the sagittal distance between the bottom of umbilicus and top of vertebra. CT measurements of the three parameters are made in triplicate by three independent operators, and the mean value is accepted for further analysis. SFT, subcutaneous fat thickness; RAT, rectus abdominis thickness; AD, abdomen depth. [file Image_1.JPEG]
